# Development and validation of a novel Context-Based Prospective Memory Task among neurotypical adults

**DOI:** 10.1590/2317-1782/20242023180en

**Published:** 2024-05-10

**Authors:** Dasmine Fraclita D’Souza, Sharon Ashley, Gagan Bajaj, Sheetal Raj Moolambally, Jayashree Sunil Bhat

**Affiliations:** 1 Department of Audiology and Speech Language Pathology, Kasturba Medical College Mangalore, Manipal Academy of Higher Education - Manipal (Karnataka), India.; 2 Department of Medicine, Kasturba Medical College Mangalore, Manipal Academy of Higher Education – Manipal (Karnataka), India.; 3 Nitte Institute of Speech and Hearing – Mangalore (Karnataka), India.

**Keywords:** Prospective Memory, Social Context, Psychometric, Assessment, Supermarket

## Abstract

**Purpose:**

To address the paucity and potential of context-based prospective memory (PM) assessment tasks suitable to Indian ethnicity, the study aimed to develop a novel context-based PM task and determine its psychometric properties among neurotypical adults.

**Methods:**

Rendered images in 2-D were extracted from a 3-D shopping mall, where PM and ongoing tasks were embedded within them to provide participants with a semi-immersive experience. The design and scoring of the novel task were constructed in alignment with the Memory for Intentions Screening Test. Fifty neurotypical adults in and around Mangaluru were recruited. The Memory of Intentions Test (MIST) and novel context-based PM task were administered.

**Results:**

The validity of the novel task was established with a Content Validity Index of 0.98. The intraclass correlation for the test-retest reliability of the novel context-based PM task was 0.92 (p<0.001) and the inter-rater reliability was 0.98 (p<0.001). The internal consistency of the six subscales was high (Cronbach’s α= 0.86), and the Spearman-Brown coefficient indicated a strong split-half reliability of 0.87. Spearman’s correlation showed that the trials exhibited strong connections to the dichotomic characteristics of the subscales to which they belonged. Further, McNemar’s test suggested similar profiles of the participants for the MIST and novel task.

**Conclusion:**

The results of the present study suggest that the novel context-based PM task offers good validity and reliability measures, providing valuable insight into the mechanisms of PM, and therefore, could be ideal for inclusion in a battery of cognitive assessments.

## INTRODUCTION

Prospective memory (PM) is the ability to recall a formed intention in the future^(1)^. Examples of PM tasks include making a phone call, sending a text, wishing someone on their birthday, passing a message on seeing somebody, and remembering to take the college identity card. PM plays a crucial role in enabling individuals to live independently by ensuring that essential tasks and responsibilities are fulfilled without constant external reminders or assistance from others, thus allowing individuals to function autonomously by effectively managing their time to accomplish the intended tasks^(2)^. Studies have reported that PM complaints are more frequent than other cognitive errors^(3)^. This may be because PM failures can have more immediate and practical consequences, such as forgetting to take medication or attending an appointment, whereas retrospective memory failures may be less noticeable in daily life. Additionally, PM complaints have been associated with poor quality of life, especially among aging adults who independently manage their essential activities of daily living and among those with cognitive impairments^(4)^. PM deficits have been reported among individuals with cognitive communicative disorders such as Mild Cognitive Impairment, Dementia, Traumatic Brain Injury, Parkinson Disease, and Autism Spectrum Disorder^(5)^

Each PM task inherently comprises a retrospective component (remembering the content of the task) and a prospective component (remembering when to execute the task)^(6)^. The entire process of PM is a result of an interplay between different cognitive processes. PM relies on attention for cue detection in the environment, working memory for intention retention, and executive control for managing attention, cue monitoring, and strategy use^(6)^. Based on the nature of the cue that triggers PM intents, PM tasks can be event- or time-based. In an event-based condition, the ability to recall a PM intention occurs when it is triggered by an external cue (e.g., remembering to fill the petrol when passing through the petrol pump). However, in a time-based condition, remembering a PM intention requires auto-initiation within a predefined timeframe (e.g., remembering to call someone at a predetermined time). Successful execution relies on holding intent in memory during ongoing activities. There are four stages involved in the performance of a PM task: formulation of intention, retention of intention, initiation of intention, and execution of intention^(7)^. The initial phase is the moment when the intent is created, which frequently involves creating a plan. In the second phase, retention of the intention is a timeframe in which the intention is stored in memory. This timeframe is typically occupied by ongoing activities that prevent continuous rehearsals in the working memory. In the third phase, the initiation of the intention is the point at which the formed intention is anticipated to be executed. In the fourth phase, the intention is executed when the anticipated PM intention is executed according to a previously formulated plan.

PM abilities, a key aspect of cognitive assessment, are commonly evaluated through self-reported questionnaires or performance-based measures. Several self-reported questionnaires have demonstrated potential for assessing PM ability or deficits, providing a subjective perspective using a Likert-type rating scale^(8)^. Performance-based measures are performed objectively by using test batteries or experimental procedures. Some of the commonly used performance-based tools for PM include the Memory for Intentions Screening Test (MIST)^(9)^, Rivermead Behavioural Memory Test-3 (RBMT-3)^(10)^, Cambridge Prospective Memory Test (CAMPROMPT)^(11)^), and Royal Prince Alfred Prospective Memory Test (RPA-ProMem)^(12)^. These performance-based measures assess PM through few standard established criteria such as event-based tasks^(9-11,13)^, time-based tasks^(9-11,13)^, an ongoing task^(9,11)^, and a 24-hour task^(9)^. These tools have demonstrated good construct validity, internal consistency, test-retest reliability, inter-rater reliability, and alternate form reliability^(9,14)^. Some of these tools have been adopted in diverse cultures^(15,16)^ and are sensitive in assessing those with brain damage, Mild Cognitive Impairment, cognitive decline, aging, other neurodegenerative disorders and neuropsychiatric conditions^(13,17-19)^

The performance-based tools described here are limited to evaluating the PM in a specific context. Context influences the encoding and retrieval of an intended action^(2)^. Therefore, when designing and administering PM tasks, it is important to consider the context to ensure the ecological validity and representation of real-life situations, as the context in which PM intentions are formed and retrieved is expected to significantly influence PM performance. During PM testing, context reduces cognitive load, aids resource allocation^(1)^. PM performance was enhanced by anticipated context^(20)^. Context is an important factor in PM retrieval in real-life settings, and there could be potential benefits of incorporating context-based elements into PM assessments to enhance their effectiveness. Previous efforts have evaluated PM within a contextual framework through tools such as the Virtual Week (VW)^(21)^, Prospective Remembering Video Procedure (PRVP)^(22)^ and Test Écologique de Mémoire Prospective (TEMP)^(23)^. The VW^(21)^ is based on a board game format, while PRVP^(22)^ and TEMP^(23)^ are in a video-based format assessing event-based PM and time-based PM. These context-based PM assessments are reported to have good psychometric properties^(24)^ demonstrating strong clinical sensitivity^(12)^, reliability^(12,22,23)^ split-half reliability^(12)^, strong internal consistency^(22,23)^, and ecological validity^(23)^.These tests are also sensitive in identifying PM deficits among people with brain damage, neurodegenerative disorders, and neuropsychiatric disturbances^(12,23)^.

Although PM abilities are an integral part of several cognitive assessment batteries, there is a significant dearth of culturally appropriate context-based PM tasks with acceptable psychometric properties in India. Relying on Western tasks poses challenges in stimuli reliability and normative data validity for the Indian population. There is a noteworthy need to develop and validate a context-based PM task among the Indian population that can address the current gap in the domain of PM assessments and provide a more dependable normative reference. Thus, the present study aimed to construct a culturally relevant context-based PM task that would also incorporate the most extensively researched and appropriate PM assessment parameters, such as types of PM intentions and scope for error analysis. The objectives of this study were to design and construct a novel context-based PM task and establish its psychometric properties among neurotypical adults.

## METHODS

### Participants

Fifty college-going young adults (18 – 25 years) [Males: N – 19, mean age – 21.1 years, SD – 2.40 years; Females: N – 31, mean age – 21.8 years, SD – 4.8 years] from various higher education institutions [Medical: N – 2, Paramedical: N – 38, Non-medical: N – 10] in and around Mangalore participated in the study. All eligible participants were required to sign and provide informed consent. The participants had a minimum qualification of 12^th^ grade with English as the medium of instruction. Participants with a Mini-Mental State Examination (MMSE) score greater than 28 were included in the study^(25)^. Individuals with normal or corrected vision and hearing sensitivity were included, while those with a presence/history of psychological or neurological deficits were excluded from the study. Prior ethical approval was obtained from the Institutional Ethics Committee (IEC KMC MLR 05-2022/165). The methodology described below describes the construction and validation of the context-based PM task administered to the study participants to establish psychometric properties. Along with the context-based PM task, each participant also underwent PM assessment on the MIST^(9)^ to establish concurrent validity with the novel context-based PM task.

### Novel context-based PM task: construct, design and protocol

The present study focused on developing a computerized PM task with event- and time-based PM targets, along with ongoing events embedded in the context of a shopping mall. To create a semi-immersive PM experience during the experiment, 3D modelling was used to construct a shopping mall that included 3D models of 20 stores or areas from a shopping mall. The 20 different stores or locations of the mall include an entrance, baggage counter, grocery shop, cutlery shop, fish shop, meat shop, bakery shop, chocolate shop, home décor shop, travel shop, electronic shop, cosmetic shop, clothing shop, footwear shop, toy shop, billing counter, food court, customer service, mall exit, and parking lot. Rendered images in a 2-D format from the two vantage points were produced for each model to obtain 40 rendered images. These 40 images were then edited to embed the ongoing PM targets.

### Ongoing task

The ongoing task in the present experiment required the participants to search for certain numbers placed on different images of the shopping mall. For example, the ongoing task in the present experiment needed participants to search for numbers comprising ‘81’ in it at any digit position (Example:76581, 812, 6814). Each image was designed to consist of 30 – 45 words or numbers, out of which at least one to six ongoing targets were possible in each image of the shopping mall. To prepare these ongoing targets, 3-to-5-digit number combinations were generated through random number generation in MS Excel. Although words were not an ongoing target in the present experiment, they were still embedded in each scene, such as numbers, to serve as distractors. For the wordlists, 11 culturally relevant semantic categories were shortlisted, and all words that might fit each category were generated. These categories include body parts, clothing, people’s names, items found in the kitchen, fruits, food dishes, electronic items, liquids, countries, places in India, and professions. Once the ongoing target was finalized, the number of targets and distractors from each number/word category that appeared in each image was determined through randomization. Ongoing stimuli were strategically placed across each image to ensure that they did not disturb the clarity and integrity of the specific scene of the mall. Microsoft PowerPoint presentation (Office 16) was used for this purpose and each ongoing stimuli was uniformly constructed with the font style ‘Calibri (body),’ a font color of ‘black’ or ‘white’ (based on the background of placement), with a font size of ‘20’ points.

### PM task

**Figure 1:**
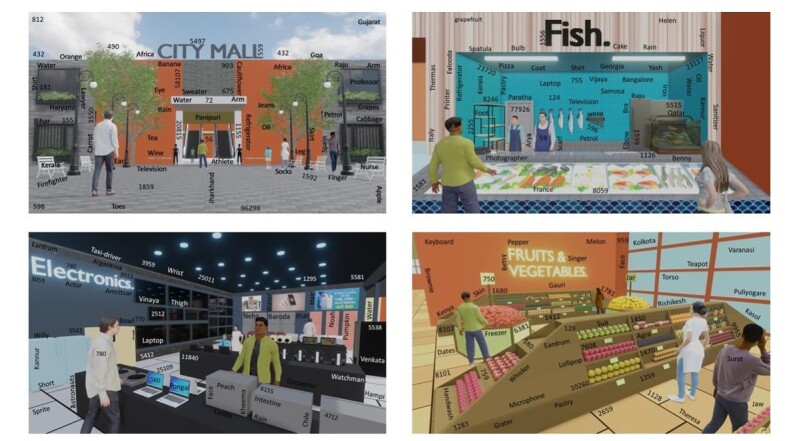
Snippets of the shopping mall

In the present experiment, the PM task was nonfocal in nature and required participants to either verbally or nonverbally perform a given action at a designated time or location in the shopping mall (Table 1). Ten PM tasks across three dichotomies, short vs. long duration tasks (time lapse between the presentation of the intention to its execution: less than vs. more than 4 min), time vs. event-based tasks (cue of the stimuli: executing an intention at a predefined time vs. location of the mall), and action vs. verbal tasks (nature of the execution: executing a verbal vs. nonverbal intention) were developed. The presentation of these PM tasks was inspired by the presentation style of MIST^(9)^ to ensure that, at any given time, the cognitive load for the participant ranged between one and four targets. Cognitive load refers to the total number of PM intentions to be remembered during the execution of a PM target. For example, during the execution of the first task, which is to ‘Ask me if I need anything from the mall in 1 minute’, the participant is holding four active PM intentions as part of their cognitive load for future execution. The description of each of the PM trials, presentation sequence, corresponding cognitive load, and affiliation of each task to the three dichotomies are presented in Table 1. A sample image of the novel context-based PM task with ongoing targets and distractors is shown in Figure 1.

**Table 1:** PM trials, the presentation sequel, and the corresponding cognitive load

Trials	Instructions	Cue	Response modality	Time delay	Order of execution	Cognitive load
1	When you come across a shop where seafood can be bought, count and tell me the number of salesmen present.	Event-based	Verbal	Short	2	3
2	Ask me if I need anything from the mall, 1 minute from now.	Time-based	Verbal	Short	1	3
3	When you are at the section where you can buy lollipops, point out any object that is green-coloured.	Event-based	Action	Long	3	4
4	After a duration of 11 minutes, let the me know the name of the store you are situated in.	Time-based	Verbal	Long	8	2
5	Write the word ‘GOLDFISH’ when you reach the section where T shirts are sold.	Event-based	Action	Long	5	3
6	After 3 minutes, write down your phone number on the sheet next to you.	Time-based	Action	Short	4	4
7	When you reach the section where you can order pizza, remember to show the examiner ‘J’ key on the keyboard.	Event-based	Action	Long	9	2
8	After 2 minutes, name 3 items you can see in the room.	Time-based	Verbal	Short	6	4
9	After 1 minute, show a thumbs up to the examiner.	Time-based	Action	Short	7	3
10	Write the name of your bank when you see a blue van.	Event-based	Action	Long	10	1

### Content validity

A modified version of the Veroy validation criteria [as mentioned in^(26)^] was used to obtain content validity for the novel context-based PM task. Five speech-language pathologists (working in the field of cognition for one or more years) and five young adults (with a profile similar to the inclusion criteria of this study) validated the content of the developed experiment with respect to clarity and instructions of items, presentation and clarity of images, size of the text, suitability of items, attainment of purpose, presentation and organization of items, scale and evaluation rating, and task duration. The items were rated on the basis of appropriateness ranging from “Not appropriate” to “Very Appropriate”. Items rated as “Not Appropriate,” “Somewhat Appropriate,” and “Can’t say” were scored 0, and items rated as “Appropriate” and “Very Appropriate” were scored 1. The content validity index was computed for these data, and all necessary attributes of the tasks were revised until the final version obtained a satisfactory content validity index.

### Procedure

The finalized version of the task was imported into SuperLab licensed version 6.0 software (Cedrus Corporation, San Pedro, CA). The duration of each image was set to 30 seconds with an inter-image interval of 3 seconds . The stimulus was presented through an ACER laptop (Swift 3) on a 14-inch screen display. The tests were conducted in a quiet environment. Each participant performed a novel context-based PM task and the MIST^(9)^. To prevent any order or sequence effects, 50% of the participants were first presented with a context-based PM task, whereas the other 50% were presented with the MIST^(9)^. The approximate time taken to administer the context-based PM task was 20 minutes. A timer was placed in front of the participants to allow them to keep track of their time. The participants were also provided with pen and paper for the tasks where they were required. Each task was verbally administered by the examiner and the participants’ responses were documented simultaneously. The participants were instructed as follows: You will be shown different scenes from the mall. Each scene contains random numbers and written words. You will have to identify numbers that have the combination ‘81’ in them (example, 7814, 481, 812). While you are doing this, I will give you certain tasks that you will have to remember to perform at the appropriate moment without prompts. You have a stopwatch in front of you to help you keep track of time to execute the required task.” The investigator noted the performance of each participant for the ongoing and PM events, along with the computation of different errors.

To demonstrate the reliability of the novel task across raters, the novel context-based PM task was simultaneously administered by two examiners to ten study participants. The examiners, trained in coding errors, were instructed to evaluate them simultaneously while they sat apart with a clear view of the participant completing the task. The test-retest reliability of the novel PM task was established by re-administering the test to ten participants after a span of two weeks.

### Scoring

Participants’ performance on the MIST^(9)^ was scored according to the test criteria and protocol. The performance of the novel PM task was computed with respect to the accuracy of the ongoing task, execution of the PM targets, and nature of the errors exhibited. Each correct identification of the ongoing target was scored as ‘1.’ A maximum score of ‘138’ could be obtained to correctly identify all the ongoing targets during the entire task. The accuracy for PM targets was scored ‘0, ‘1’ or ‘2’ for the time-based tasks and ‘0’ or ‘2’ for the event-based tasks. An errorless and prompt PM execution yielded a score of ‘2’ whereas a PM performance with a misplaced timing (+/- 15 secs) was scored as ‘1.’ PM failure was scored as ‘0.’ Six subscale scores were computed based on dichotomies described earlier. The first dichotomy was based on task execution delays. Task numbers 1, 2, 6, 8, and 9 and task numbers 3, 4, 5, 7, and 10 assessed PM within short and long time delays, respectively. The second dichotomy was based on the nature of the cue that triggered the response. Tasks 2, 4, 6, 8, and 9 and tasks 1, 3, 5, 7, and 10 assessed time-based triggers and event-based triggers, respectively. The third dichotomy assessed PM based on the nature of the responses. Tasks 1, 2, 4, and 8 assessed verbal-based responses, and tasks 3, 5, 6, 7, 9, and 10 assessed action-based responses. The total PM score was computed by adding all subscale scores, making it equal to 60.

Furthermore, the nature of the errors exhibited by the participants was recorded and categorized according to error classification, followed by the MIST^(9)^. The first error code was prospective memory failure (PF). It was scored when the participant failed to respond to a PM trigger. The second error code, task substitution (TS) error, was scored if participants responded correctly but substituted a task from a different prompt. The loss of content (LC) error, the third error code, was coded when a participant acknowledged a PM cue but seemed to have forgotten all or part of the task. The fourth error code, loss of time (LT) error, occurred when a correct task was carried out at the incorrect time, and random error (RE) was coded when a response could not be classified as any other error. The frequency of each error for all participants in the novel task and the MIST^(9)^ was computed.

### Data analysis

Jamovi software (2.3.21) and IBM SPSS version 25 were used for the statistical analysis. The mean and standard deviation were determined using descriptive statistics for each of the subscales of the MIST^(9)^ and the novel context-based PM task. The convergent validity of the novel context-based PM task with the MIST^(9)^ performance was established using McNemar’s test. Cronbach’s alpha and Spearman-Brown’s split-half reliability were used to determine the internal consistency within the subscales. The intraclass correlation coefficient (ICC) was used to analyze the test-retest and inter-rater reliability of the PM scores of the context-based PM task as well as their respective error codes.

## RESULTS

The novel context-based PM task had six subscales based on the duration aspects of the trials (short and long), cue-based aspects of the trials (time-based and event-based), and modality of response of the trials (verbal and action). The total scores of all subscales yielded the total PM score for each participant on the task. The descriptive statistics of the subscale-specific and total performance of young adults in the novel context-based PM task are presented in Table 2.

**Table 2:** Mean, median, and standard deviation of the subscales wise and total performance on MIST and context-based PM task

Subscale	Maximum		Mean		Median		SD	
	MIST	CBPMT	MIST	CBPMT	MIST	CBPMT	MIST	CBPMT
Short duration task	10	8	7.88	7.1	8	8	1.44	1.2
Long duration task	10	8	5.36	5.42	6	6	2.13	1.68
Time-based task	10	8	7.24	5.64	7	6	1.6	1.47
Event-based task	10	8	6	6.88	6	8	2.42	1.57
Verbal response task	10	8	5.24	6.98	5	8	1.45	1.33
Action response task	10	8	8	5.54	8	6	2.21	1.79
Total score	60	48	39.7	37.6	42	40.5	8.79	6.89

**Caption:** SD = Standard Deviation; MIST = Memory for Intentions Screening Test; CBPMT = Context-based Prospective Memory task

Five error codes were used to code the types of errors made by participants during the task. The percentage of each error type that occurred during the task is listed in Table 3.

**Table 3:** Percentage of occurrence of each error type in the PM trials of context-based PM task and MIST

CBPMT							MIST						
Trial	PF	TS	LC	LT	RE	None	Trial	PF	TS	LC	LT	RE	None
1	8	0	2	22	0	68	1	2	0	4	0	0	94
2	20	0	12	0	0	68	2	4	6	18	0	0	72
3	12	0	24	0	0	64	3	4	4	0	2	0	90
4	8	6	6	28	0	52	4	10	6	14	4	0	66
5	18	18	12	2	2	48	5	10	0	2	0	0	88
6	10	0	8	16	2	64	6	2	6	8	0	0	84
7	0	0	0	8	0	92	7	4	0	2	2	0	92
8	46	12	18	6	0	18	8	36	8	20	10	0	26
9	52	2	8	4	2	32							
10	8	2	4	2	0	84							

**Caption:** PF = Prospective memory failure; TS = Task substitution; LC = Loss of content; LT = Loss of time; RE = Random error, MIST = Memory of Intention Screening Test; CBPMT = Context-Based Prospective Memory Task

### Consistency and reliability

To indicate how closely related the set of tasks was to one another, Cronbach’s alpha and Spearman-Brown split half reliability were administered to the context-based PM task. High internal consistency was observed across the six subscales (Cronbach’s α= 0.86). The Spearman-Brown’s coefficient for the six subscales was 0.87 indicating a strong split half reliability. Furthermore, Spearman’s correlation was used to determine the association between performance on each trial and the subscale scores. The findings revealed a statistically significant association (as indicated in Table 4) between performance in each trial and the respective subscale scores, except for the action response of the seventh trial.

**Table 4:** Spearman’s rank correlation between the performance on each trial and the subscale scores

Task	Duration		Cue		Response modality		Prospective Memory Total
	Short Delay	Long Delay	Time based	Event based	Verbal Response	Action Response	
1	0.456*	0.134	0.383	0.099	0.314	0.179	0.310
2	0.501*	0.246	-0.211	0.647*	0.526*	0.196	0.391
3	0.212	0.523*	0.172	0.508*	0.17	0.573*	0.506*
4	0.316	0.241	0.535*	0.055	0.038	0.425	0.318
5	0.106	0.382	0.061	0.401	0.108	0.399	0.309
6	0.432	0.05	0.596*	-0.174	0.423	0.016	0.238
7	0.282	0.023	0.334	-0.111	0.131	0.08	0.111
8	0.092	0.367	0.653*	-0.02	0.509*	0.139	0.354
9	0.143	0.500*	0.061	0.497*	0.248	0.475*	0.482*
10	0.054	0.567*	-0.054	0.558*	0.089	0.548*	0.455*

* p<0.05

To establish inter-rater reliability, PM performance in each trial, along with the coding of errors in these trials, was independently performed by two assessors for 20% of the total sample size (n=10). An interclass correlation coefficient (ICC) of 0.98 {with a 95% confidence interval from 0.92 to 0.99 (F (9,9)= 47.25, p<0.001)} and 0.89 {with a 95% confidence interval from 0.58 to 0.97 (F(9,9)= 9, p<0.005} was obtained for the trial performance and error codes between the two raters, respectively. These results indicated good inter-rater reliability for the novel context-based PM task.

By administering the task again (after two weeks), the test-retest reliability of the task was determined to be 20% of the total sample size (n=10). An ICC of 0.92 {with a 95% confidence interval, 0.71 to 0.98 (F(9,9)= 13.022, p<0.001)} for the trial performance was obtained suggesting good test-retest reliability. Additionally, ICC of 0.78 {with a 95% confidence interval, 0.17 to 0.95 (F(9,9)= 4.556, p>0.005)} was obtained for the error codes, indicating good test-retest reliability.

Five speech-language pathologists with expertise in cognition and five young adults validated the content of the novel task using the modified content validation checklist developed by Veroy^(26)^. Table 5 presents the results and criteria used to validate the context-based PM task.

**Table 5:** Relevance rating on the items scale by 5 experts and 5 young adults

Items	Experts					Young Adults					No. of Agreement	I-CVI	UA
	1	2	3	4	5	1	2	3	4	5			
Clarity and instructions of items	1	1	1	1	1	1	1	1	1	1	10	1	1
Presentation and clarity of images	1	1	0	1	1	1	1	1	1	1	9	0.9	0
Size of the text	1	1	1	1	1	1	1	1	1	1	10	1	1
Suitability of items	1	1	1	1	1	1	1	1	1	1	10	1	1
Attainment of purpose	1	1	1	1	1	1	1	1	1	1	10	1	1
Presentation and organization of items	1	1	1	1	1	1	1	1	1	1	10	1	1
Scale and evaluation rating	1	1	1	1	1	1	1	1	1	1	10	1	1
Task duration	1	0	1	1	1	1	1	1	1	1	9	0.9	0
											**S-CVI/Ave**	0.98	
Proportion relevance	1	0.88	0.88	1	1	1	1	1	1	1	**S-CVI/UA**		0.88
Average proportion of items judged as relevant across the 5 experts and 5 young adults: 0.98													

Caption: I-CVI = item-level content validity index; S-CVI/Ave = scale-level content validity index based on average method; S-CVA/UA = scale-level content validity index based on universal agreement method; UA = universal agreement

### Convergent validity

Participants’ performance on the novel context-based PM task was evaluated to determine its convergent validity. For this, the performance of the participants in the context-based PM task was matched with their performance on the Memory of Intentions Test (MIST)^(9)^. The subscale-wise and overall performance of the participants in the MIST^(9)^ and error codes are shown in Table 3.

Quartile distribution was performed on the total performance scores obtained by the participants on the novel context-based PM task and the MIST^(9)^. The quartile allocation of a given participant in the MIST^(9)^ was associated with the quartile allocation of the context-based PM task using McNemar’s test. The results of McNemar’s test suggested that the proportion of participants in both groups across the four quartiles was not statistically different (p=0.411). Furthermore, the Spearman correlation for different types of errors by the participants was obtained from the MIST^(9)^ and context-based PM tasks. The percentage of errors observed in the MIST^(9)^ had a strong positive correlation with the percentage of errors observed in the context-based PM task (r=0.94, p<0.05). Hence, the findings of both tests indicated that the context-based task had strong convergent validity with the MIST^(9)^ scores in determining the performance levels and nature of the PM errors.

## DISCUSSION

Having realized the paucity of context-based PM assessment tasks suitable for the Indian ethnicity, the present study aimed to develop a context-based PM task and establish its psychometric properties among young adults. This study chose to construct a PM task in the context of a shopping mall for its diverse locations, engagement opportunities, and naturalistic environment that aids task recall through environmental cues. Other studies have also reported that familiarity with the shopping mall setting offers greater relatability and comfort for people of all age groups^(27)^. Ouellet^(28)^ highlighted the benefits of utilizing memory evaluation centered on a virtual shopping environment (The Virtual Shop), where individuals are required to navigate through a diverse array of stores and complete prospective memory tasks within specific stores. The importance of contextualizing memory evaluations to real-world encounters has been highlighted earlier, including activities such as driving down a street^(22)^, navigating through a shopping district^(29)^, or executing memory tasks in specific target locations^(28)^.

The PM and ongoing stimuli considered in this study were developed in a sophisticated manner using 3-D models to depict the minor intricacies that are a part of the shopping mall. Each item or object in the 3-D model was arranged in a rational manner such that none of them distorted the overall coherence of the scenes. The appropriateness of the construct of these designs was further reinforced by five experts and five young adults who provided a content validity index of 0.98 regarding the clarity and instructions of items, presentation, clarity of images, size of the text, suitability of items, attainment of purpose, presentation and organization of items, scale and evaluation rating, and task duration.

For a PM assessment task to be holistic, it is important to incorporate different categories of PM tasks. For example, in contrast to RBMT-3^(10)^, CAMPROPMT^(11)^ and RPA-ProMem^(13)^ which lacked certain elements of PM assessment, MIST^(9)^ offers a comprehensive assessment of various PM parameters, surpassing all previously proposed test batteries. Given this holistic approach to PM assessment, the current study mapped PM trials to the dichotomies proposed by MIST^(9)^ to ensure that the novel context-based PM task can offer multiple attributes of PM. Thus, the present study designed the PM trials across these dichotomies and segregated the performance based on these subscales. Additionally, the novel context-based PM task also provided PM assessments across a varying degree of cognitive load, where the number of items to be remembered during any execution was dynamic and captured while evaluating PM. Similar importance regarding cognitive load has been incorporated in other tests like MIST^(9)^.

While assessing PM, the ongoing task is often not emphasized. It is crucial to include an ongoing activity alongside the PM target task, particularly if researchers wish to evaluate the PM that replicates real-world conditions. Intentions, such as remembering to take medication at a certain time or to attend an appointment, often coincide with ongoing tasks. Ongoing tasks in assessments simulate real-life distractions, highlighting potential performance errors and strategic insights in PM. To enhance realism and engagement, context-based PM assessments with ongoing tasks are valuable, given the multitasking nature of the real-world. However, current PM assessments vary in evaluating PM performance and incorporating ongoing tasks, with some tests considering them and others neglecting their impact on PM performance. The present study addresses this by integrating an ongoing task to measure accuracy. Although the study did not explore the ongoing accuracy, the novel context-based PM task can be implemented without the PM task establishing a basis for comparing PM and ongoing performance, thereby determining the ongoing cost which is the decline in the performance of the ongoing task owing to an increase in the PM load^(30)^.

Furthermore, the present study also investigated the nature of erroneous responses, like the error rating approach used by MIST^(9)^. PM errors were classified into five error types: loss of content (LC), loss of time (LT), task substitution (TS), PM failure (PF), and random error (RE). Error analysis helps not only identify error types but also correlate them with stages of the PM process. As mentioned earlier, the PM process occurs in four stages: formation of intention, retention of intention, initiation of intention, and execution of intention^(7)^. AN LC error suggests a problem in the second level of PM processing indicating failure to store (encode) intention until execution. An LT error can occur at the intention initiation level, where the intention content is retained but there is failure in time monitoring. A TS error indicates a breakdown at the PM execution phase, where the intention is initiated but substituted with an alternative task. This might be due to ongoing task interference or insufficient monitoring, often from forgetting the correct sequence. A PF error is complete intention loss during retrieval, causing PM execution failure. This error can occur for various reasons, such as insufficient encoding or retention of the intention in memory or interference from ongoing tasks that may distract the individual from retrieving the intention at the appropriate time. Occasionally, an RE can occur when there is an issue in any phase of the PM process.

Internal consistency was measured for the novel context-based PM task to ensure that the items in the test measured the same construct. Cronbach's alpha 0.86 of the novel context-based PM task indicated good internal consistency. To further assess the reliability of the novel context-based PM tasks, the split-half reliability of the six subscales was performed, which revealed a split-half correlation coefficient of 0.83. While determining the relationship between the trials and all subscales, it was observed that the correlation was not linear. The reason for such non-linearity was acceptable because the trials showed strong connections only with the dichotomous characteristic of the subscales to which they belonged. For example, if trial 2 was short delay event-based verbal task, high correlations were seen towards the subscale scores of ‘short-delay,’ ‘event-based’ and ‘verbal modality’ respectively, while the subscale scores of ‘long-delay,’ ‘time-based’ and ‘action’ modality’ did not correlate due to their dichotomic differences. Hence, it was determined that only trials mapped to their corresponding dichotomies provided a high correlation. Furthermore, test-retest reliability, which measures the consistency or stability of a test over time, was assessed, and a score of 0.92 was obtained, indicating good consistency, was obtained if this test had to be repeated by an individual over time. Inter-rater reliability was used to assess the degree of agreement or consistency between two or more examiners on whether they were assessing or scoring the task in a consistent manner. The novel context-based PM task's interrater reliability was 0.98, confirming the task's strong reliability as a tool for measuring PM, irrespective of the raters. In addition to the PM accuracy scores, the error codes also showed good test-retest and inter-rater reliability coefficients of 0.78 and 0.89 respectively. Thus, the novel context-based PM task, along with its sound and robust psychometric properties, provides a holistic evaluation of PM abilities, including assessing PM accuracy, ongoing accuracy, PM cost, ongoing cost, subscale analysis, and error analysis.

The study was also interested in exploring how well the performance of the novel context-based PM task could be related to MIST performance. Performing a direct correlation may not serve its purpose because of the inherent differences in the tasks with respect to their nature of presentation, focality, context, and ongoing task. Hence, the similarity in a quartile-based distribution on the performance of the participants in the MIST and the novel task was studied. The findings of McNemar’s test found no significant differences in the quartile-based profiles of the individuals across both tests, suggesting that a high performer was identified as a high performer on both measures and vice versa for low performers. Furthermore, Spearman's correlation for the various error categories identified among the participants across both tasks was found to be 0.94, reinforcing the current tool's strong convergent validity. The preliminary results indicate that the newly developed context-based PM tasks possess favorable psychometric qualities that enable a better understanding of prospective memory mechanisms.

### Limitations and future directions

This study can be extended to evaluate the psychometric properties of the novel task among individuals from different age groups, such as middle-aged and elderly individuals, across various educational levels. In the real world, individuals rely heavily on external aids; however, the current test assesses the reliability of their internal mechanisms, which is comparatively challenging. Additionally, it would be interesting to explore the various strategies people deploy to accomplish PM in the present task and compare their performance-based metrics with perception-based metrics. By addressing these limitations through further research, the novel context-based PM task could become an essential component of cognitive-communicative assessment batteries. Once the psychometric characteristics are firmly established within the context of the typically aging population, future studies could evaluate the discriminant validity and other psychometric functions among individuals with a wide range of cognitive-communicative disorders.

## CONCLUSION

This study aimed to develop and establish the psychometric features of a novel context-based PM task among neurotypicals. The novel context-based PM task was constructed to deliver a semi-immersive experience of a shopping mall using 2D rendered images from 3D modelling and was constructed with reference to the assessment parameters used in MIST^(9)^. The findings of this study identified the novel context-based PM task as a valid and reliable measure of PM among neurotypical young adults. The present study highlights the importance of including this task in cognitive communication assessment batteries and supports its further exploration in other typical and clinical populations.
